# Development of a Machine Learning Classifier for Brain Tumors Diagnosis Based on DNA Methylation Profile

**DOI:** 10.3389/fbinf.2021.744345

**Published:** 2021-11-08

**Authors:** Yuxing Chen, Yixin Yan, Moping Xu, Wen Chen, Jinyu Lin, Yan Zhao, Junze Wu, Xianlong Wang

**Affiliations:** ^1^ Department of Bioinformatics, School of Basic Medical Sciences, School of Medical Technology and Engineering, Key Laboratory of Medical Bioinformatics, Key Laboratory of Ministry of Education for Gastrointestinal Cancer, Fujian Medical University, Fuzhou, China; ^2^ Fujian Stomatological Hospital, Fujian Medical University, Fuzhou, China

**Keywords:** machine learning, multilayer perceptron model, CNS tumors, DNA methylation, classification

## Abstract

**Background:** More than 150 types of brain tumors have been documented. Accurate diagnosis is important for making appropriate therapeutic decisions in treating the diseases. The goal of this study is to develop a DNA methylation profile-based classifier to accurately identify various kinds of brain tumors.

**Methods:** Thirteen datasets of DNA methylation profiles were downloaded from the Gene Expression Omnibus (GEO) database, of which GSE90496 and GSE109379 were used as the training set and the validation set, respectively, and the remaining 11 sets were used as the independent test set. The random forest algorithm was used to select the CpG sites based on the importance of the features and a multilayer perceptron (MLP) model was trained to classify the samples. Deconvolution with the debCAM package was used to explore the cellular composition difference among tumors.

**Results:** From training datasets with 2,801 samples, 396,568 CpG sites were retained after preprocessing, of which 767 were selected as the modeling features. A three-layer MLP model was developed, which consists of 1,320 nodes in the hidden layer, to predict the histological types of brain tumors. The prediction accuracy is 99.2, 87.0, and 96.58%, respectively, on the training, validation and test sets. The results of deconvolution analysis showed that the cell proportions of different tumor subtypes were different, and it is approximately enough to distinguish different tumor entities.

**Conclusion:** We developed a classifier that is robust for the classification of central nervous system tumors, and tried to analyze the reasons for the classification performance.

## Introduction

Brain cancer is an umbrella term accounting for many malignant tumors affecting different tissues in the nervous system. Survival rates for brain tumors vary widely, depending on the type of tumors and other factors. Recently, the classification of brain tumors has been evolving rapidly. The fourth edition of the World Health Organization (WHO) classification, published in 2016, introduced molecular parameters for the first time in addition to histology to define many tumor entities. Despite an enormous advancement of the 2016 classification system which facilitates the clinical, experimental and epidemiological studies that would lead to improvements in the lives of patients with brain tumors, it has raised many concerns and been considered outdated at the time of publication. It has been superseded by the 5th edition (WHO CNS5) that was released recently.

During the last decade, next-generation sequencing (NGS) and other high-throughput molecular profiling techniques supplying comprehensive data further transformed the diagnosis of brain tumors and the advances in machine-learning provided more accurate prediction of outcome and response to therapy. Among different omics, DNA-methylome profiling has been proven a valuable technique that can complement the diagnostic process, in particular for histologically ambiguous tumors.

DNA methylation is an epigenetic mechanism involving the binding of a methyl group onto the C5 position of the cytosine *via* a covalent bond (Portela and Esteller, 2010). DNA methylation regulates gene expression by recruiting proteins involved in gene repression or by inhibiting the binding of transcription factor(s) to DNA (Zafon et al., 2019). Similar as transcriptomics, DNA methylation provides rich information on the molecular characteristics of cells. But the high stability of the covalent modification makes it ideal for clinical diagnosis and applicable to formalin-fixed and paraffin-embedded specimens, circumventing the stringent sample preparation requirements in mRNA profiling (Koch et al., 2018).

DNA methylation-based biomarkers have been used in diagnosis and prognosis for brain tumors. For instance, hypermethylation of MGMT promoter in glioblastoma indicates that the tumor is sensitive to temozolomide treatment, though the predictive capability is limited to the IDH wild-type gliomas (Reifenberger et al., 2017). Chen et al. (2017) developed a prognostic signature for in IDH mutant glioblastoma based on the expression levels of 10 glycolytic genes, and found that these genes are hypermethylated in the promoter region in patients with better prognosis, demonstrating a potential to use the methylation statuses of the promoter regions of these genes for prognosis. Based on DNA methylation profiles, ependymomas were clustered into nine subgroups, which reflects different locations, genomic and epigenomic characteristics of the tumors. Similarly, choroid plexus tumors were divided into three subgroups based on DNA methylation, one of which is located in the fourth ventricle of adults and the other two occur mainly in children (Thomas et al., 2016; Pienkowska et al., 2019). Machine learning algorithms have been applied in the classification of brain tumors using DNA methylation levels as features. Zhang et al. (2020) adopted support vector machine (SVM) and other algorithms to develop classifiers for cell lines from 13 tissues including brain, lung and others, and the classification accuracy reached to 96.3%. Gomez et al. (2018) developed two diagnosis classifiers with the linear discriminant analysis method, which were used to distinguish four subtypes of medulloblastomas and the accuracy rates reached to 99 and 92%, respectively. Therefore, machine-learning algorithms demonstrate a great potential in developing signatures for classification, prognosis and prediction of brain tumors based on the whole genome DNA methylation profiles.

Recently, Crapper et al. (2018) developed a random forest classifier to improve pathological diagnosis of nearly 100 brain tumor entities based on genome-wide DNA methylation patterns and demonstrated its application in a routine diagnostic setting. Later, they reported a DNA-methylation-based diagnostic model for practical diagnostic scenarios. In the validation set, the classes of 12% of the samples could not be predicted due to insufficient prediction scores, and only 1% of the samples were predicted incorrectly. However, the classifier needs 10,000 DNA methylation probes as input features, which make it liable to the overfitting risk and difficult to translate the classifier into a clinical detection kit. In this study, we aim to develop an accurate and robust classifier for brain tumors using fewer probes in order to improve clinical translatability and interpretability of the machine learning model.

## Materials and Methods

### Data Collection

We downloaded 13 DNA methylation datasets of brain tumors in the raw data format from the Gene Expression Omnibus (GEO) database (Supplementary Table S1). Among them, GSE90496, consisting of 2,801 samples and covering 82 types of brain tumors and nine types of normal brain tissues, was used as the training set. GSE109379, consisting of 1,104 samples and covering 69 types of brain tumors, was used as the validation set. The remaining data was used as the independent test set, which consists of 1,200 samples and covers 17 types of brain tumors. The distribution of the data sets is shown in Figure 2. All the datasets were generated by the Illumina Infinium HumanMethylation450 BeadChip (450k) and Infinium MethylationEPIC BeadChip (850k).

### Data Preprocessing

The data preprocessing was carried out with the R language (v. 4.0.0). Using the minfi (Aryee et al., 2014) (v.1.34.0) package to import the IDAT files, we performed background correction (bg.correct function) and dye correction (correct the average probe intensity to 10,000) for the two channels, and then performed batch effect correction to correct the difference between formalin-fixed paraffin-embedded (FFPE) and freshly frozen samples and/or other systematic bias on the log2 converted fluorescence intensity values (removeBatchEffect function, limma (Ritchie et al., 2015) package (v.3.24.15). Estimated batch effects were also used to correct the validation set and test set. Beta values were calculated from the retransformed intensities using an offset of 100.

In addition, we deleted the following probes 1) probes located on the sex chromosomes, 2) probes that cannot be uniquely aligned to the hg19 reference genome, 3) probes containing single nucleotide polymorphisms, 4) probes not included in the EPIC chip. The remained 396,568 probes were used for downstream analysis.

### Feature Selection

The feature selection and model development were carried out with the *Python* language (v.3.8.3) with the scikit-learn (Pedregosa et al., 2011) module (v.0.23.1). The selection of the probes for model development was based on their importance coefficients in the random forest model which was optimized iteratively for the hyperparameters and number of probes. The initial values for the random forest model include “random_state” = 0, “n_estimators” = 100, “max_features” = “sqrt” and “class_weight” = “balanced’.” The RandomForestClassifier function was used to fit the training set to the model and an importance coefficient was assigned to each probe.

A set of top ranked probes were selected to develop the multilayer perceptron (MLP) model and to optimize the hyperparameters in order to achieve the highest accuracy in the validation set. With the optimized hyperparameters, the importance coefficients were updated with the new fitting model. This iteration was repeated manually to obtain a minimal set of features without sacrificing the accuracy too much. In the final model, 767 probes were selected for modeling.

### Multilayer Perceptron Model Development

A MLP model is a class of feedforward artificial neural network consisting of three types of layers, including input, hidden and output layers. A minimal 3-layer model was built in this work to avoid the potential overfitting problem. The model was trained using the MLPClassifier function from the scikit-learn module. The hyperparameters of the model were optimized along the features selection as mentioned in the previous section. The hidden layer consists of 1,320 nodes in the final model and the activation method is Rectified Linear Unit. The model was solved using the random gradient descent method with the adaptive learning rate and a maximum number of iterations of 1,000.

### Deconvolution Analysis

First, the random forest model was applied to select top 10,000 probes based on their importance coefficients assigned by fitting the model to the training dataset. Next, with the debCAM (Chen, 2020) package (v1.6.0), an unsupervised and reference-free deconvolution was performed on the beta values profiles of the 10,000 probes. The number of subpopulations, aka. cell components with distinct DNA methylation features, was limited from 2 to 20, and debCAM determined the optimal number to be 10. Cell types were identified using marker genes from CellMarker database (Zhang et al., 2019).

### Statistics and Plotting

The heatmaps were drawn with the ComplexHeatmap package (Gu et al., 2016) (v.2.6.2) in R. The probability density curves were plotted with seaborn’s kdeplot function (Waskom, 2021) (v.0.11.0) in python. The nonlinear dimensionality reduction algorithm, *t*-Distributed Stochastic Neighbor Embedding (*t*-SNE), was carried out using the TSNE function from scikit-learn with the default parameters. Other statistical analysis and plotting were performed with R (v.4.0.0).

## Results

### Identifying Methylation Markers With Classification Capability by Random Forest

**FIGURE 1:**
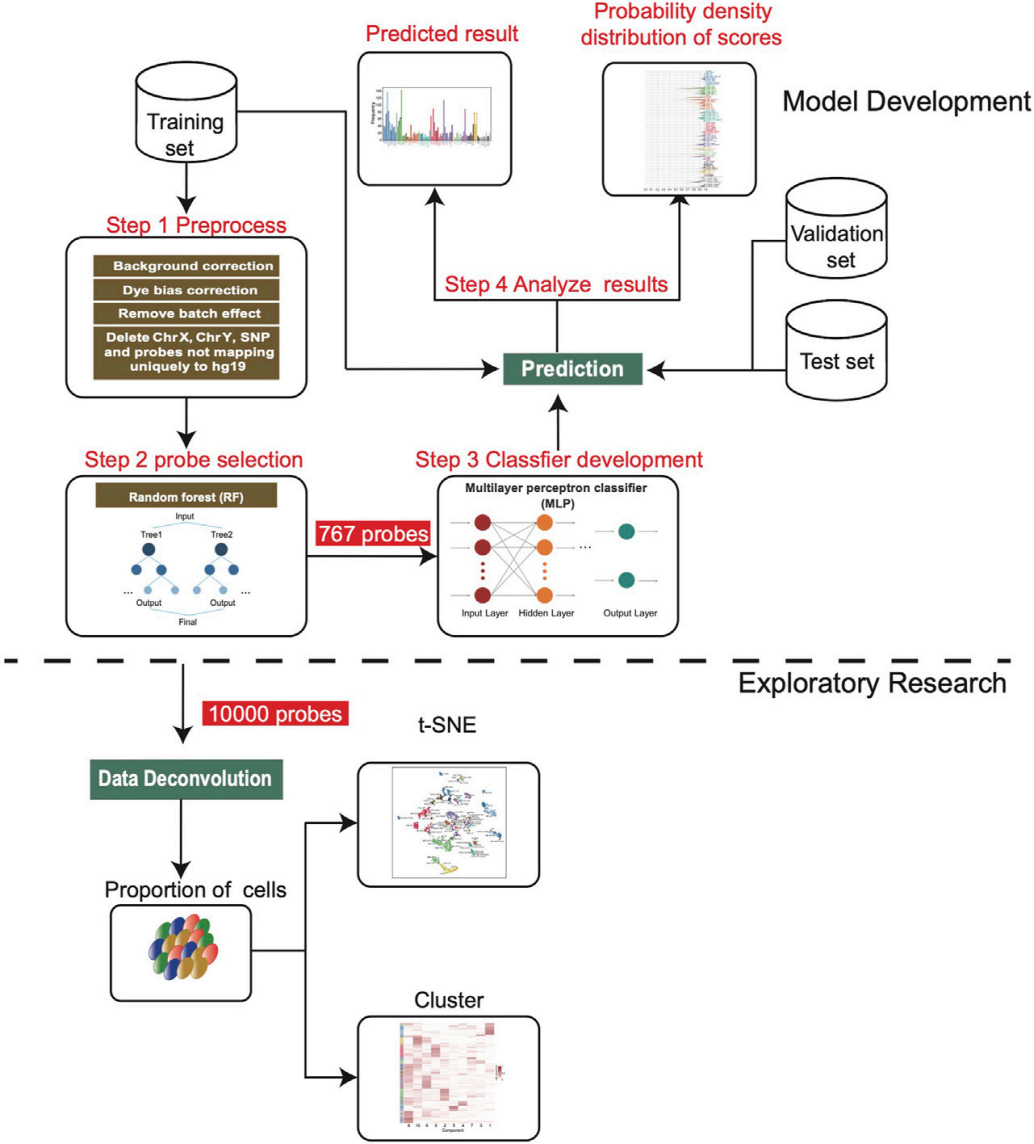
Workflow chart of this study. After the training set was preprocessed, we screened 767 features to develop the MLP model, and then applied it on the validation set and independent test set. In addition, 10,000 features were screened for deconvolution analysis to estimate the cell composition of each sample.

**FIGURE 2:**
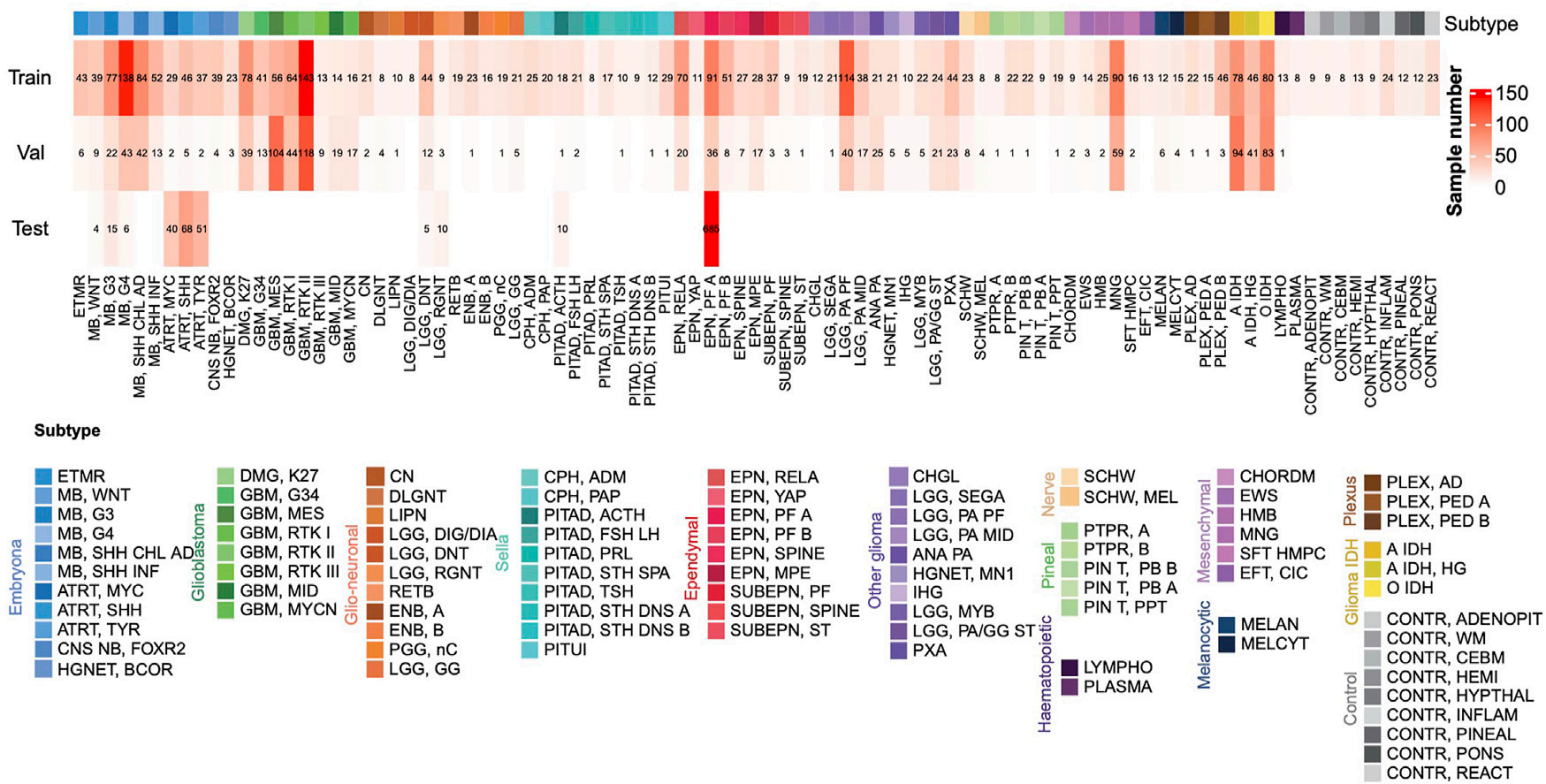
The numbers of samples of different subtypes in the training set, validation set and test set. The color annotation corresponding to each subtype is consistent in this article.

The flowchart of this study is shown in Figure 1. We downloaded 13 sets of DNA methylation profiles of various types of brain tumors measured by the Illumina 450k and EPIC (850k) microarray platforms from the GEO database (Figure 2), of which GSE90496 was used as the training set, GSE109379 as the validation set, and the remaining as the independent test set (Supplementary Table S1). The training set covers 82 types of brain tumors and nine types of normal control tissues located in different brain regions, with a total size of 2,801 samples. The validation set contains 69 types of brain tumors with a total sample size of 1,104 and the independent test set consisting of 1,200 samples covers 17 types of brain tumors (Figure 2).

All the original methylation profiles were preprocessed with the same procedure to obtain the beta value matrices as detailed in the Materials and Methods section. In particular, the batch effect correction formulas obtained in the training dataset were saved and latter applied to individual profiles in the validation and test tests to ensure that the same transformation rules were used for all the samples. In total, 396,568 probes remained after preprocessing with a standard deviation greater than 2.62 × 10^−3^ across 2,801 samples in the training dataset.

**FIGURE 3:**
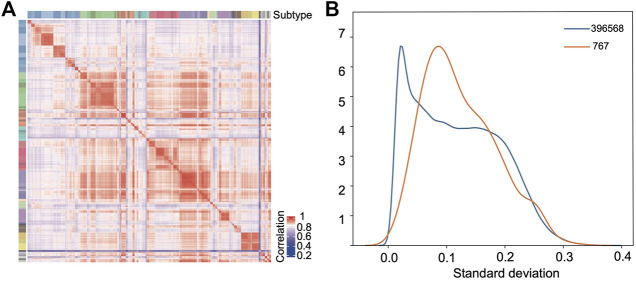
Selected features show strong classification capability. **(A)** Pearson correlation heatmap based on the selected probes among the samples in the training set. **(B)** Distribution density curves of the standard deviations of all the probes (blue) after preprocessing and the selected 767 probes (orange).

The random forest model was used to further select the probes as the modeling features based on their importance coefficients. The importance coefficient represents the information gain associated with the addition of the feature to the model, the larger the coefficient, the greater the classification ability of the feature. The feature selection and the hyperparameters optimization were carried out iteratively as detailed in the Materials and Methods section. In the final model, 767 probes were selected which let the classifier reach the highest accuracy in the validation set. The distribution of the importance coefficients of these probes is shown in Supplementary Figure S1. Using the beta values of these probes to calculate the Pearson correlation between samples as the similarity measure, hierarchy clustering of the samples in the training set showed that they clustered together according to their subtypes (Figure 3A), which demonstrates the feasibility for classification of the selected features.

Figure 3B compares the empirical probability density distribution of the standard deviations of the 767 features across samples with that of all probes. From Figure 3B, we see that although the selected probes tend to have a larger standard deviation than the background probes, half of the probes still have very low standard deviations (*s* < 0.1). Therefore, the selection method based on standard deviations would miss these probes which have consistently higher (or lower) beta values in the samples of one class with a limited size and consistently lower (or higher) beta values in all other classes of samples.

### Performance Evaluation of 3-Layer Perceptron Model in the Training and Validation Datasets

**FIGURE 4:**
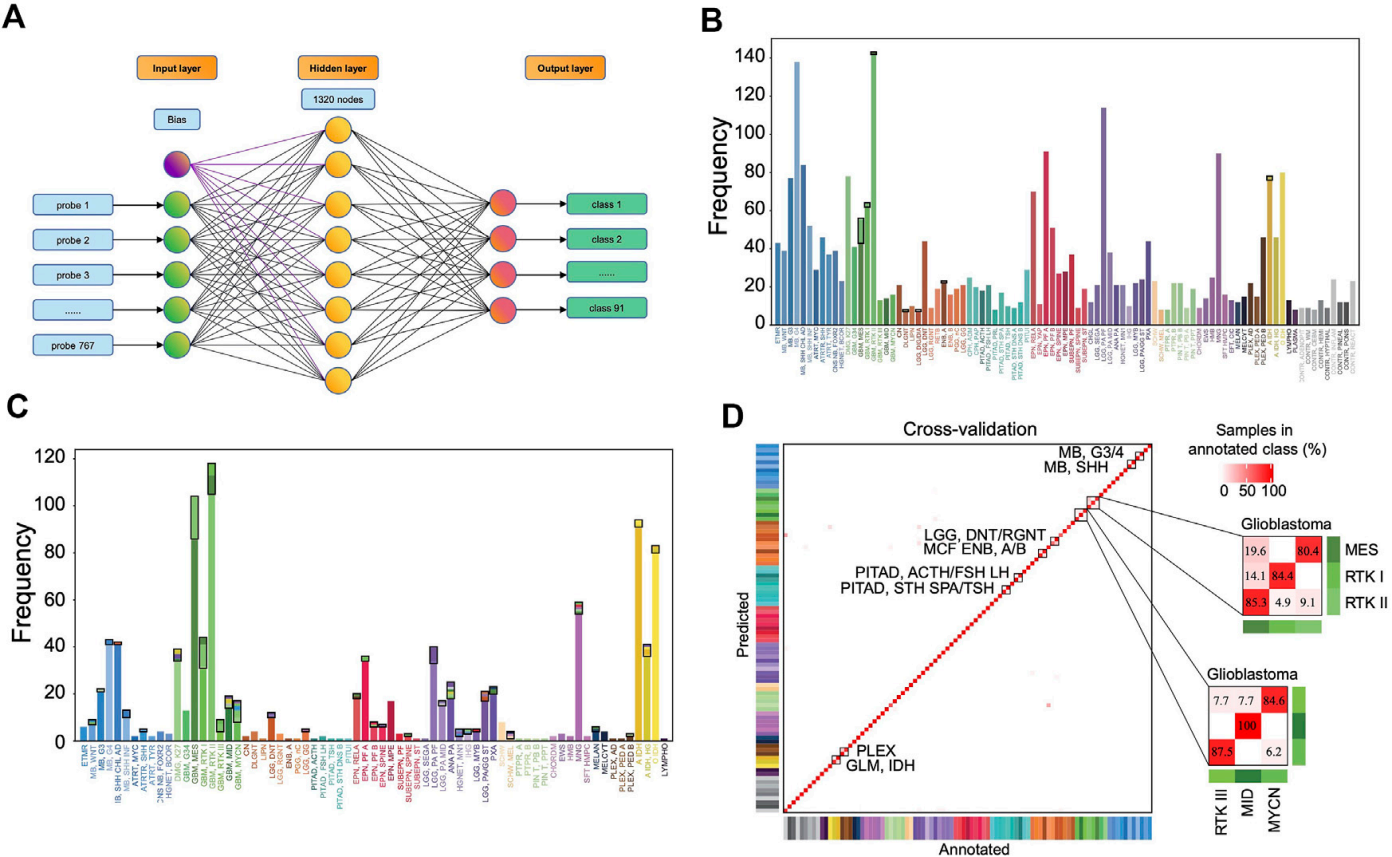
Classification performance of the MLP model. **(A)** The three-layer perceptron model structure. The input layer consists of 767 nodes, corresponding to 767 features. The hidden layer has 1,320 nodes, and the output layer has 91 nodes corresponding to 91 classes. Distribution of the prediction results in the training set **(B)** and in the validation set **(C)**. Each column represents one tumor class. The bottom parts represent the correctly predicted results and the top parts enclosed by a black rectangle represent the incorrect results where the colors correspond to the predicted classes. **(D)** Heat map showing results of a 5-fold cross-validation of the MLP classifier in the training set, which incorporates the result allocated to 91 methylation classes of all 5-iterations. The diagonal data cells represent classification accuracies while the off-diagonal cells represent the misclassified rates. For example, 45 of the 56 “GBM, MES” samples were correctly classified (80.4%), and 11 samples were misclassified as “GBM, RTK II” (19.6%). The color of annotation and abbreviations can refer to Figure 2. Methylation class families (MCF) are indicated by black squares.

A 3-layer perceptron model was developed for classification (Figure 4A), in which the input and output layers consist of 767 and 91 nodes, respectively, while the hidden layer consists of 1,320 nodes in the optimized model. The activation function is the rectified linear unit function, and the output given a sample input is a vector of 91 values with a sum of 1, which correspond to the predicted probabilities for the 91 classes. The class with the largest probability is considered as the predicted result.

The accuracy rate of the classifier is 99.2% in the training set and 87.0% in the validation set (Figures 4B,C). The latter is much higher than the performance of the random forest classifier (accuracy = 79.1%) in the validation set developed with the same set of features. In the training set, 5-fold cross-validation showed an accuracy of 0.961, 0.966, 0.957, 0.968, and 0.952, respectively, in five iterations. The results showed that most errors occur in the subtype assignment of glioblastomas (GBM) (Figure 4D). What is particularly worth noticing is that a few subtypes form a cluster, such that “GBM, MES,” “GBM, RTK I” and “GBM, RTK II” forming one class and “GBM, RTK III,” “GBM, MID” and “GBM, MYCN” forming another class. Most misclassification cases occur within the classes. For example, 13 cases of “GBM, MES” are predicted to be “GBM, RTK II” and 2 cases of “GBM, RTK I” are predicted to be “GBM, RTK II.” The rest of the errors are distributed in other 7 types of brain tumors (Table 1). In the validation set, there are 79 samples that were incorrectly classified (Supplementary Table S2). Similarly, most of them are glioblastomas, 18 cases of “GBM, MES” predicted to be “GBM, RTK II,” 10 cases of “GBM, RTK I” predicted to be “GBM, RTK II,” and 8 cases “GBM, RTK II” predicted to be “GBM, MES.” The results suggest that different subtypes of glioblastomas have vague boundaries, which makes it difficult for the classifier to predict accurately.

**TABLE 1:** The incorrect prediction result in the training set. The first column is the true label, the second column is the predicted result, and the third column is the number of the corresponding samples.

Ground truth	Predicted Class	Count
A IDH	A IDH, HG	2
DLGNT	O IDH	1
ENB, A	ENB, B	1
GBM, MES	GBM, RTK II	13
GBM, RTK I	GBM, RTK II	2
GBM, RTK II	GBM, RTK I	1
LGG, DIG/DIA	CONTR, REACT	1

**FIGURE 5:**
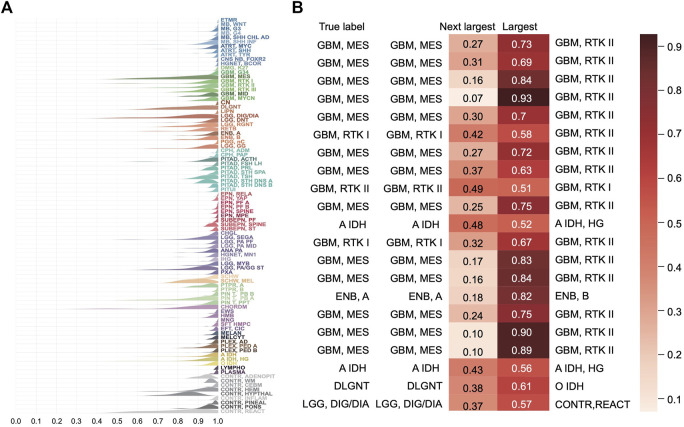
Distribution of the predicted scores for each class in the training set. **(A)** Each row represents a probability density curve of the predicted scores for each class to which the samples belong. The heights of curves were normalized. **(B)** The predicted scores of the true classes and the largest predicted scores of the incorrectly classified samples in the training set.

The distribution of the predicted class probabilities is plotted for each subtype in Figure 5A. Most of the probabilities are close to 1 and the proportions of the values less than 0.5 are nearly zero in most classes. Supplementary Figure S3A shows the distribution of the differences between the largest probabilities (predicted class probabilities) and the next largest probabilities. From Supplementary Figure S2A, we see that most of the differences are higher than 0.8 and only nine samples have a difference less than 0.1. Supplementary Figure S2B shows the differences between the largest predicted probability value and the next largest predicted probability value of the samples that were incorrectly predicted in the training set. It can be found that the distribution tends to be on the left, indicating that the model’s prediction results for these samples are not confident enough. And in fact, the classes with the next-largest probabilities are actually the true labels (Figure 5B). These results show that the classification model is sufficiently stable.

### Performance of the Multilayer Perceptron Classifier Evaluated in the Independent Test Set

Eleven data sets were downloaded from GEO, forming the independent test set, which consist of 17 subtypes of brain tumors with the histopathological labels, a total of 1,200 samples. The prediction accuracy of the MLP model on this test set is 96.58% (Supplementary Figure S3). A total of 26 samples were misclassified (Table 2). Except for a few cases, the errors occurred between similar subtypes of the same brain tumor types like GBM. For example, “GBM, RTK I” or “GBM, RTK II” were assigned to “GBM, RTK III.”

**TABLE 2:** The incorrect prediction result in the independent test set. The first column is the true label, the second column is the predicted result, and the third column is the number of the corresponding samples.

Ground truth	Predicted Class	Count
ATRT, MYC	ATRT, SHH	2
PITAD, ACTH	PITAD, FSH LH	1
PITAD, ACTH	CONTR, ADENOPIT	1
ETMR	CONTR, HEMI	1
ETMR	DMG, K27	1
GBM, MYCN	MB, SHH CHL AD	1
GBM, RTK I	GBM, RTK III	2
GBM, RTK II	GBM, RTK III	14
GBM, RTK II	LGG, MYB	1
GBM, RTK II	GBM, MYCN	2

### Cellular Components are Able to Classify Brain Tumors

**FIGURE 6:**
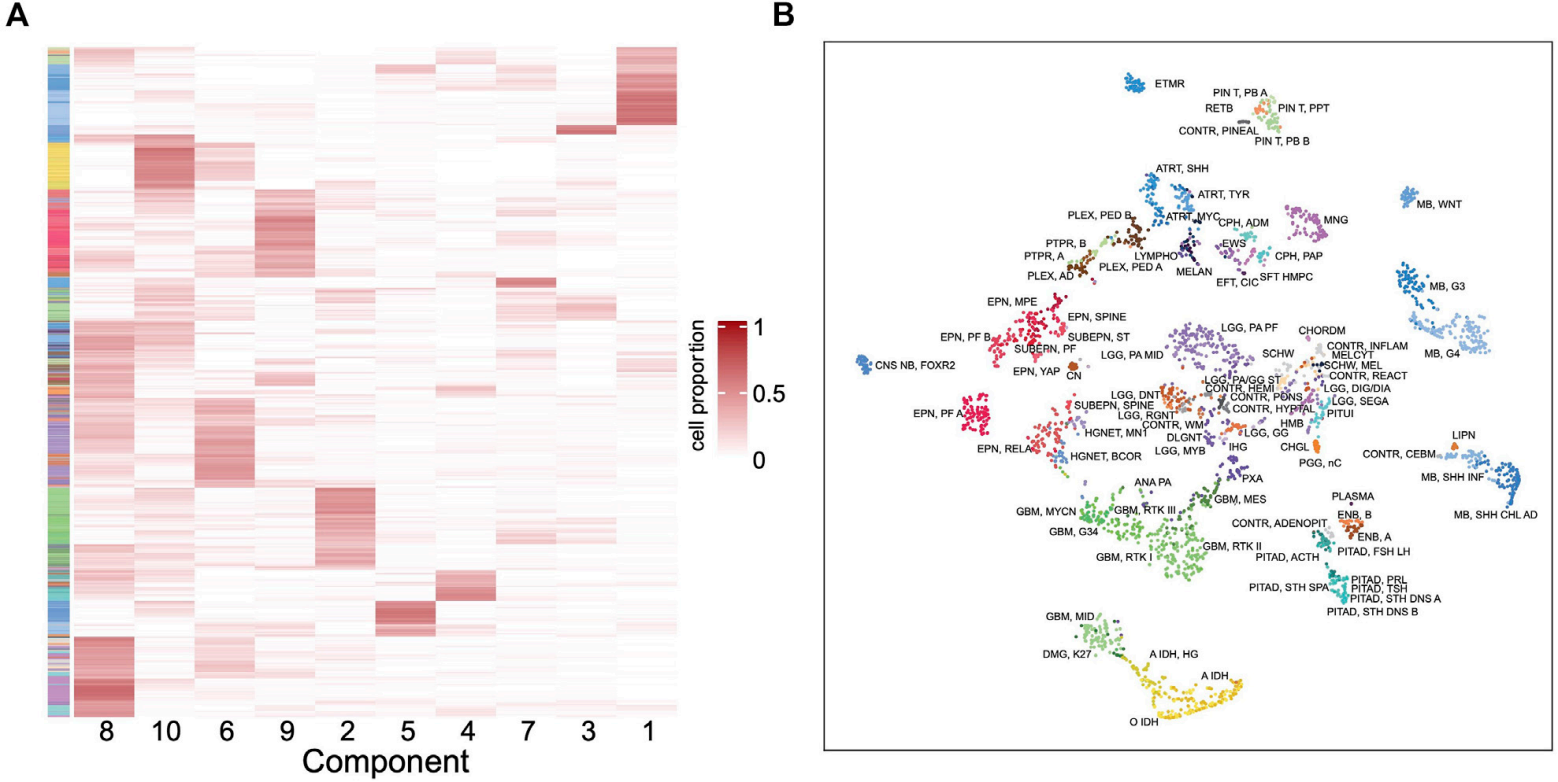
Deconvolution result in the training set. **(A)** Heatmap of the cellular proportions of 10 components in all the samples. Rows are samples and columns are the components. **(B)** The *t*-SNE dimensionality reduction result based on the proportions of 10 components.

To further understand the outstanding performance of the classification model, a reference-free deconvolution analysis was performed on the training dataset. Since the tumors are located in different brain regions, it is likely that they are composed with different cell populations and each cell type may carry unique epigenetic features. Using the random forest model, we screened top 10,000 probes with the highest importance coefficients. The deconvolution analysis was performed with these features on the training dataset using the debCAM package and 10 cell types and their proportions in each sample were obtained. A cluster analysis on the cellular proportions from the deconvolution result found that samples of the same subtype are approximately clustered together (Figure 6A). Similar results were obtained from the *t*-SNE dimensionality reduction analysis (Figure 6B). This shows that the result of deconvolution is reliable. In addition, several cell types have strong specificity, appearing in specific subtypes only, which implies that the difference in the ratio of different cell types may be the reason of a good performance of the classifier.

After mapping the methylation probes to genes and then using cell marker genes from the CellMarker database (Zhang et al., 2019), we tentatively assigned a few components to cancer stem cell, neural stem cell, Th1 cell, AXL + SIGLEC6+ dendritic cell, epithelial cell, granulosa cell, meiotic prophase fetal germ cell and mitotic arrest phase fetal germ cell. However, not every cell component has basis genes overlapping with the cell markers. These need further studies to illustrate the components and their different roles in each tumor type.

## Discussion

Currently, more than 150 types of brain tumors have been documented^1^ and the main diagnosis methods are imaging tests, including MRI and CT. Interoperative pathological examination is often needed to determine the nature of the tumors. A stereotactic needle biopsy may be done if the tumor is in hard-to-reach areas or very sensitive areas that might be damaged by a more extensive operation (Jackson et al., 2001; Villena Martín et al., 2020). More accurate classification is key to deliver precision therapy for better prognosis of patients. DNA methylation is considered to be an ideal material for tumor diagnosis and classification because the information can be stored stably in routine clinical samples (Koch et al., 2018). There are a few machine learning methods based on DNA methylation profiles that were developed to subtype brain tumors. Zhang et al. (2020) developed a classification model with SVM and other algorithms for 13 cell lines from brain, lung and other tissues, and the accuracy reached to 0.963. Gomez et al. (2018) adopted linear discriminant analysis to develop two models classifying the four subtypes of medulloblastomas with accuracy rates of 99 and 92%, respectively. The study carried out by Crapper et al. (2018) is the most comprehensive one. A random forest classifier was built, which incorporates 10,000 probes as features from Illumina’s 450k microarray platform, to distinguish 82 types of brain tumors. Performance of the model has been validated through multi-center clinical application. But we suspect that so many features may make the classifier potentially at risk of overfitting and it also make difficult to develop a fast detection kit for clinical translation. Here we built a three-layer MLP classifier with only 767 features without significant performance loss. Furthermore, the deconvolution study showed that the difference in the cellular composition is sufficient to discriminate different subtypes to a good degree.

The feature selection in the current study was based on the feature importance computed in the random forest model which measures the information gain upon addition of a feature, the larger the importance coefficient, the greater the classification ability. For multiple-category classification, it prefers the features that can distinguish many categories simultaneously and these features should have large variance across samples. But for those features with strong correlation (this is the case in DNA methylation where the methylation levels of many CpG sites are strongly correlated), only one of them would be selected. This is an advantage on one hand since the redundancy is removed, but on the other hand not necessarily the best feature is selected. The top 20% features with the largest variance are in this category, which have a median importance coefficient of 1.0E-3, ranging from 1.6E-4 to 2.5E-3 and a median variance of 3.9E-2, ranging from 2.5E-3 to 1.1E-1. Most of the rest features can only distinguish one or two classes from the other classes, so the overall variance is limited (see Figure 3B). Among them, there are two scenarios. One is hypermethylated in most classes but hypomethylated in one class and the other is opposite. The sites on the left and in the middle in Supplementary Figure S4 belong to these two scenarios, respectively. The feature selection based on the importance coefficient is biased towards the classes with larger sample sizes. In Crapper et al. (2018)’s study, they screened 10,000 probes, and our research selected 767 probes which should be a subset of Crapper’s set. Regarding the distribution of importance coefficients of the probes, there are mainly three distinct ranges (Supplementary Figure S1). The importance coefficient ranges from 0 to 6.24E-4, while the most important 767 probes are distributed between 1.29E-4 and 6.24E-4, covering 79% of the range; the most important 10,000 probes are distributed between 3.03E-5 and 6.24E-4, covering 95% of the distribution range, and the remaining probes are only distributed in a very limited importance range. Overall, it is reasonable to select 767 probes which captures the most important distinction capability.

The distribution of the probes’ genetic locations is shown in the Supplementary Figure S5A. In total, 406 probes (373 + 33) are located in the coding regions while the rest are located in the regulatory regions. Using functional enrichment analysis, we found that most pathways, enriched by the genes in which the probes are located, are related with functions of neuron cells and nervous system (Supplementary Figure S5B). Among the selected features, some methylation sites have been studied as markers or therapeutic targets in brain tumors. Probe cg11890453, located in the 5′UTR region of *SOCS6*, is hypomethylated in medulloblastoma. Tanaka et al. (2014) found interferon-β inhibited the viability of medulloblastoma and glioblastoma cells by upregulating the expression of *SOCS6*. Probe cg13257371, in the gene body region of *MSI2*, is hypermethylated in medulloblastoma. Cox et al. (2013) found that medulloblastoma cells was significantly inhibited by knocking out the *MSI2* gene. Probe cg14483244, in the gene body of *HDAC5*, is hypermethylated in the G4 subgroup of medulloblastoma, in which *HDAC5* has a higher expression than in the SHH and WNT subgroups (Milde et al., 2010). Probe cg20585869 is in the transcription initiation position (TSS) of *NEFM*. It is hypermethylated in glioblastoma which leads to a low expression of *NEFM* (Lee et al., 2015). A few genes have been included in the WHO CNS5 classification guideline (Louis et al., 2021)*.* For example, TERT (cg19977628, gene body region) promoter mutation is a biomarker in classifying “Oligodendroglima, IDH-mutant,” “1p/19q-codeleted Glioblastoma,” “Glioblastoma, IDH wildtype” and “Meningioma.” FGFR (cg14733725, cg07250222, TSS1500 region) is a marker gene in “polymorphous low-grade neuroepithelial tumor of the young”; SMO (cg01475577, gene body region) and GLI2 (cg14773228, gene body region) are marker genes in “Medulloblastoma, SHH-activated”; DICER1 (cg18503,758, gene body region) is a marker in “Embryonal tumor with multilayered rosettes.”

The complexity of brain tumors makes the classification difficult. The boundaries between different classes are often blurry. From Figure 5 and Supplementary Figure S2, we see that for the misclassified samples, the predicted classes with the second highest probability are often the true classes. In combination with histology, the predicted classes are similar with the real classes. For example, some grade II tumors tend to progress to more malignant tumors, low-grade diffuse astrocytomas may transform into anaplastic astrocytoma and glioblastoma and similar transformation occurs in oligodendroglioma and oligoastrocytomas (Louis et al., 2007). Therefore, some histological classification may be inaccurate at all as Crapper’s study found. Most classification errors are found between subtypes of GBMs which reflects that our current understanding on GBM is not deep enough. On one hand, accuracy of clinical diagnosis of GBM is only 80% (Forst et al., 2014). On the other hand, the subgroup classification of GBM is constantly changing. For example, in recent WHO CNS5 classification (Louis et al., 2021), “GBM, IDH-mutant” subtype was removed (astrocytoma IDH-mutant subtype was introduced) and pediatric-type neoplasm was excluded from GBM. For these tumors, we may combine DNA methylation classification results with genetic mutation status given in the classification guide to further improve the diagnosis accuracy.

Although an accuracy of 96.58% was obtained in the independent test set in this study, we have to admit that the dataset only included 17 subtypes and there were a large number of EPN and PF A (*n* = 685) that were easily distinguishable, so the results were not conclusive. Some subtypes have fewer data sets in public database, and more comprehensive testing is needed for future studies.

Through dimensionality reduction with *t*-SNE analysis of the cell ratio matrix obtained by the cellular deconvolution of the DNA methylation profiles in the training set, we found that the same subtypes were clustered together. This result indicates that difference in cellular composition is a major contributing factor to the profile differences among various subtypes. It is likely that the variation in cellular composition contribute significantly to the performance of the classifier, although the classifier development and profile deconvolution have different goals. This also exposes another challenge in developing the classifier based on the DNA methylation profiles. For a specific tumor entity with the same oncogenic molecular alterations, the cellular composition may vary dramatically because of different tumor microenvironment while different tumor entities may share similar microenvironment leading to the same molecular profiles. Therefore, differences in tumor microenvironment bring additional difficulties to cancer classification. Clinical relevance of the classification results should be evaluated based on the prognostic and predictive value. In the follow-up research, we will try to determine the cell components in the deconvolution results, to investigate the influence of the tumor microenvironment on the classifier, and to further improve the model’s performance.

## Data Availability

The original contributions presented in the study are included in the article/Supplementary Material, further inquiries can be directed to the corresponding author.

## Footnotes

1 American Association of Neurological Surgeons. https://www.aans.org/Patients/Neurosurgical-Conditions-and-Treatments/Brain-Tumors [Accessed April 23, 2021].
