# Supplementary figures and images for: Development of a Machine Learning Classifier for Brain Tumors Diagnosis Based on DNA Methylation Profile
Source: Front Bioinform. 2021 Nov 8;1:744345. doi: 10.3389/fbinf.2021.744345 (PMC9581020; doi:10.3389/fbinf.2021.744345)

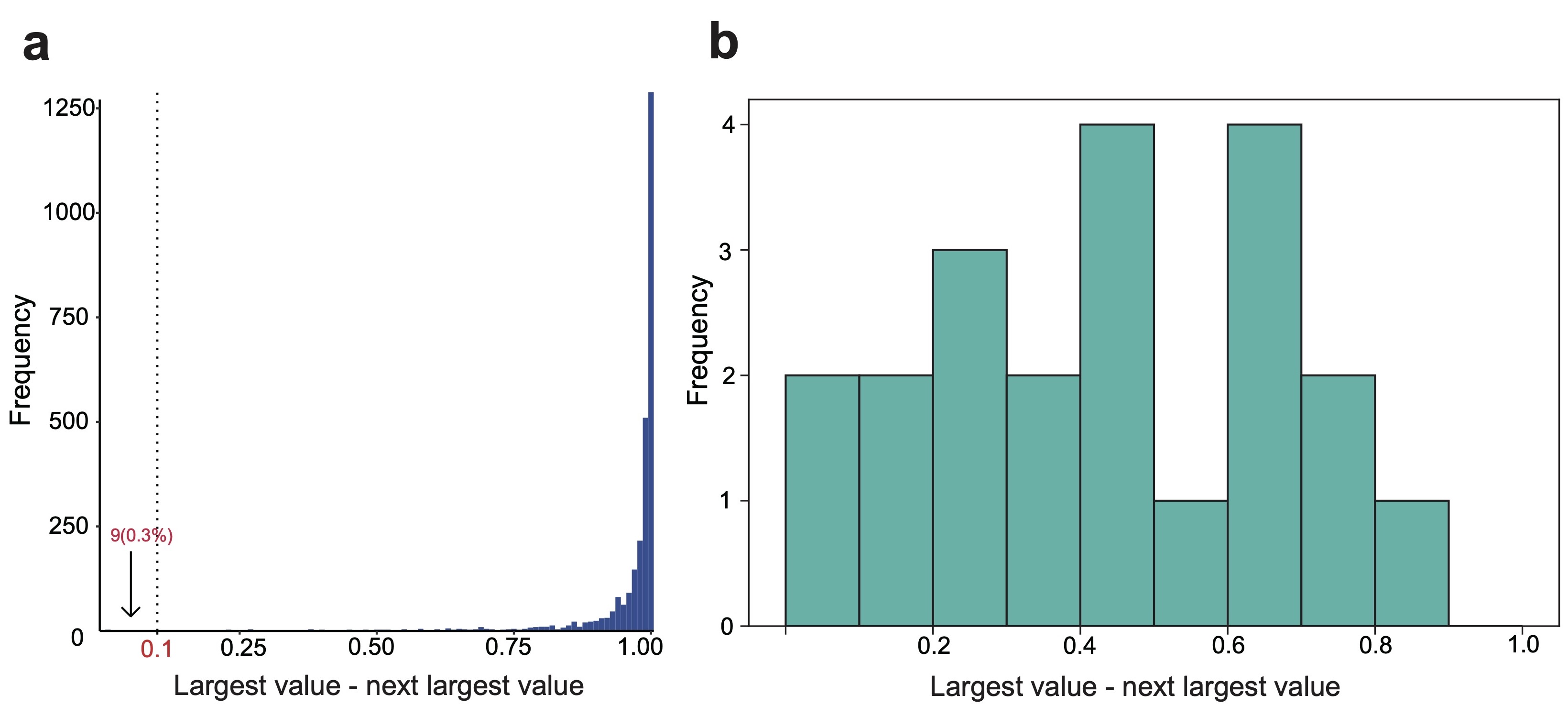

Supplement: Supplementary file 2 [file Image3.JPEG]

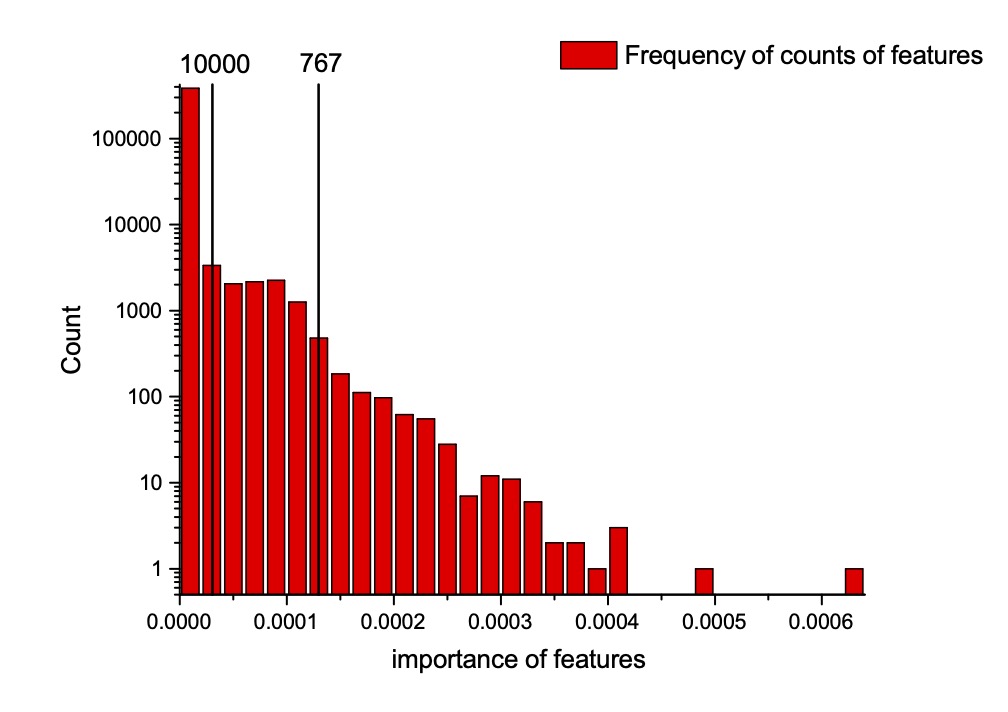

Supplement: Supplementary file 3 [file Image1.JPEG]

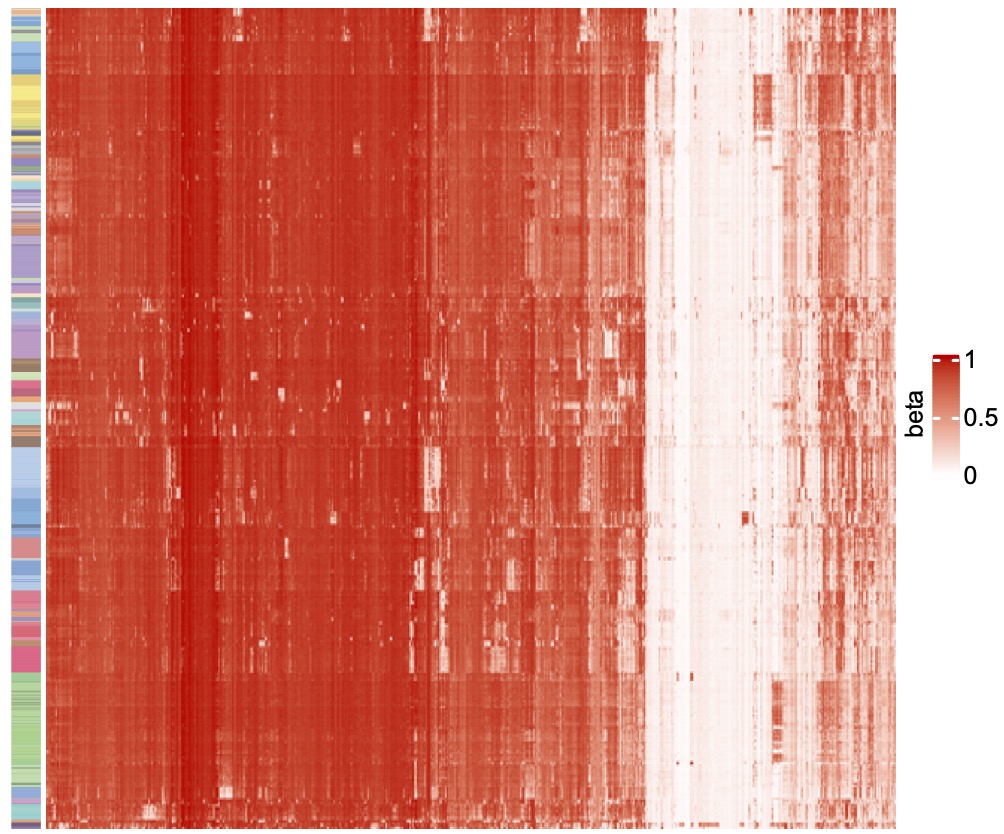

Supplement: Supplementary file 4 [file Image4.JPEG]

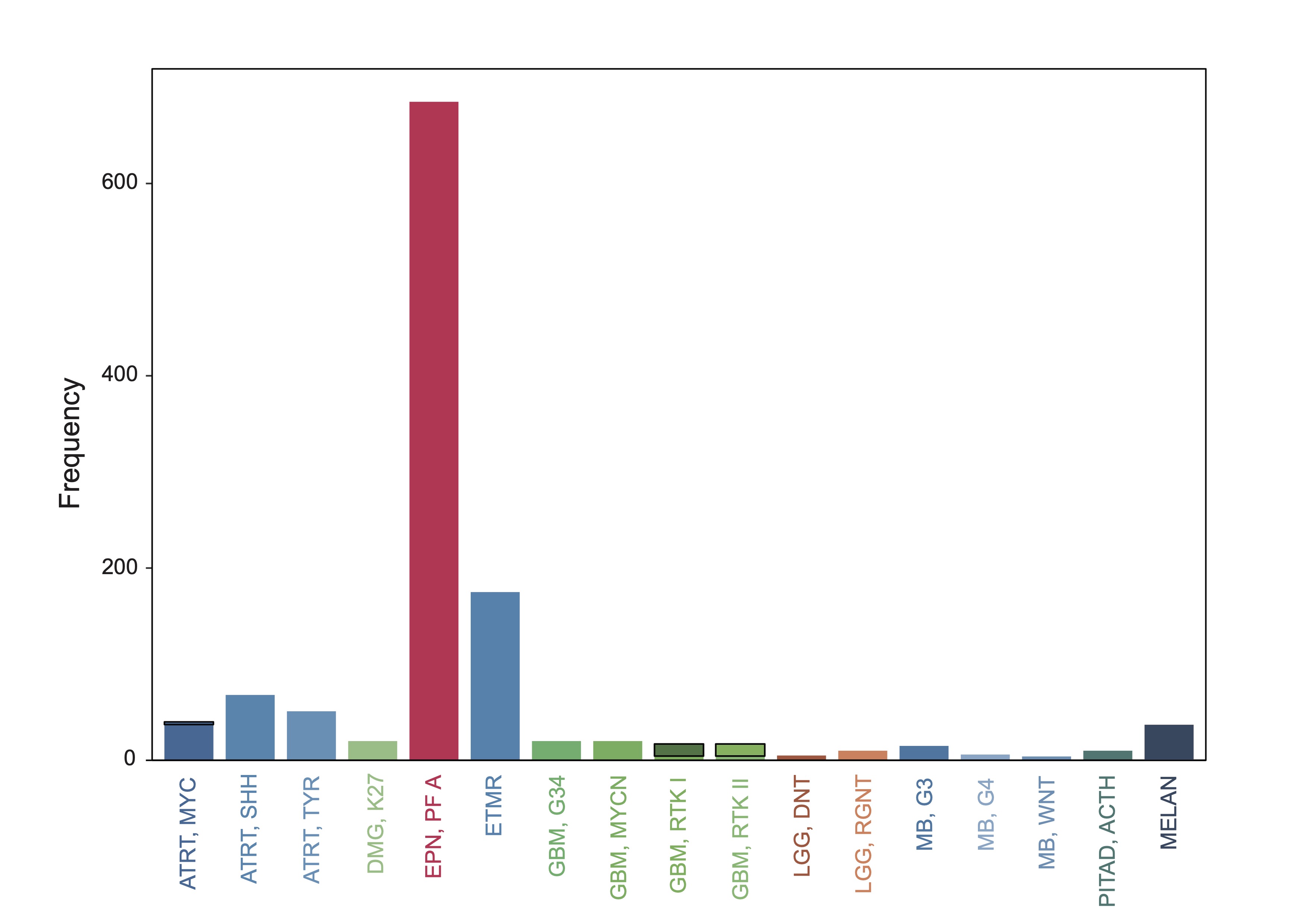

Supplement: Supplementary file 5 [file Image2.JPEG]

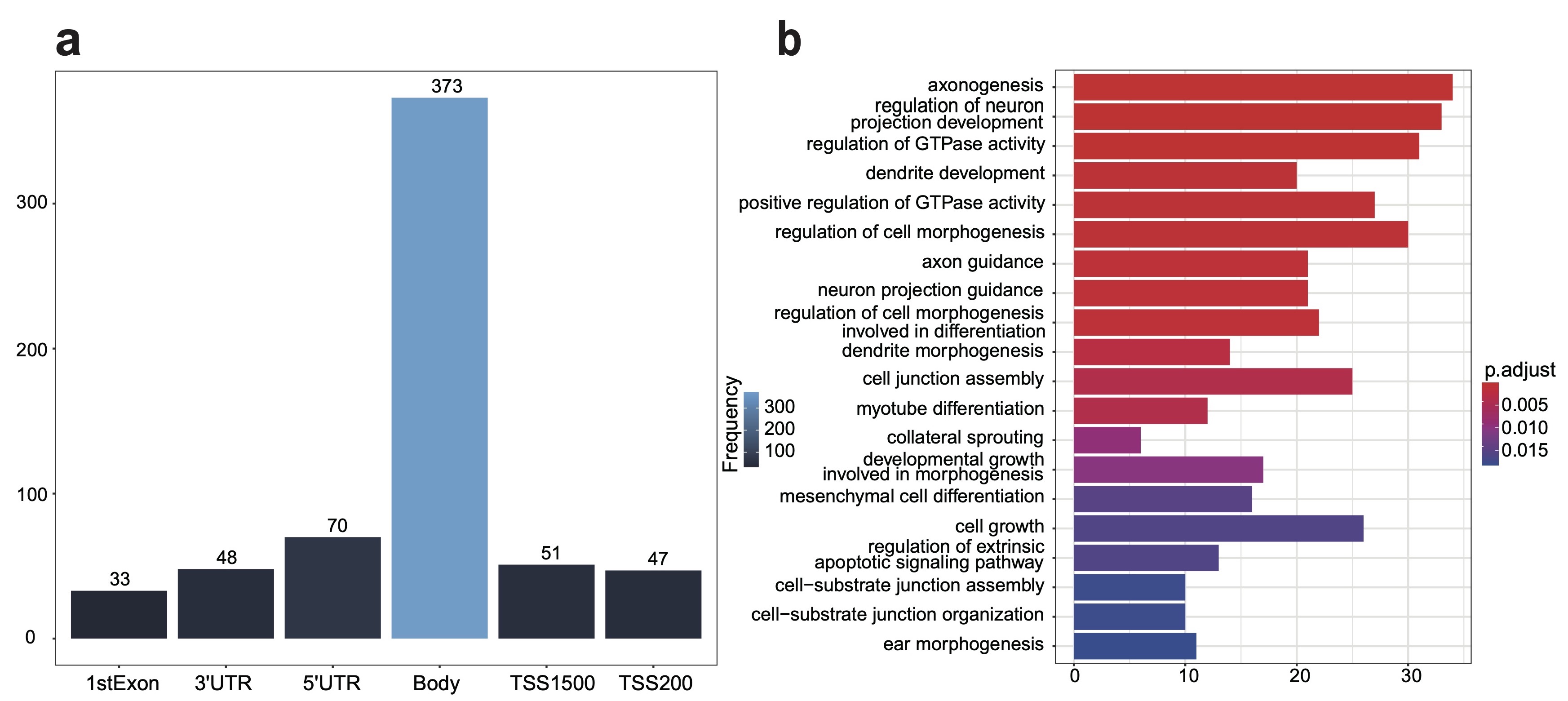

Supplement: Supplementary file 6 [file Image5.JPEG]
