# Supplementary material for: Development of a Machine Learning Classifier for Brain Tumors Diagnosis Based on DNA Methylation Profile
Source: Front Bioinform. 2021 Nov 8;1:744345. doi: 10.3389/fbinf.2021.744345 (PMC9581020; doi:10.3389/fbinf.2021.744345)
Supplement: Supplementary file 8 [file DataSheet1.DOCX]

Supplementary Figures and Tables

**Figure Captions**

Figure S1. Distribution of the importance coefficients assigned by the random forest model of all probes after data preprocessing. Two vertical lines denote the thresholds to choose top 767 probes and top 10000 probes.

Figure S2. Distribution of the differences between the largest predicted scores and the second largest predicted scores for all the samples in the training set (**a**) and for the samples that were incorrectly classified (**b**).

Figure S3. Distribution of the prediction results in the independent test set. Each column represents one tumor class. The bottom parts represent the correctly predicted results and the top parts enclosed by a black rectangle represent the incorrect results where the colors correspond to the predicted classes.

Figure S4. Heatmap of the beta values of the selected probes of the samples in the training set. Rows are samples and columns are the feature probes which were hierarchically clustered but the dendrogram is not displayed.

Figure S5. Genetic locations and functions of the selected features. (**a**) Distribution of the regions of the 767 probes on genes. (**b**) The GO pathways significantly enriched with the genes of the probes.

**Table Captions**

Table S1. Datasets for training set, validation and independent test set.

Table S2. Misclassified result of the validation set. “Ground truth” is the true label, “predicted result” is the class label misclassified by the MLP model, and “count” shows the number of misclassified samples in the corresponding category.
